# Emergent Inpatient Administration of Casirivimab and Imdevimab Antibody Cocktail for the Treatment of COVID-19 Pneumonia

**DOI:** 10.7759/cureus.15280

**Published:** 2021-05-27

**Authors:** Alexander T Phan, Janet Gukasyan, Sarkis Arabian, Sharon Wang, Michael M Neeki

**Affiliations:** 1 Internal Medicine, Arrowhead Regional Medical Center, Colton, USA; 2 Critical Care, Arrowhead Regional Medical Center, Colton, USA; 3 Infectious Disease, Arrowhead Regional Medical Center, Colton, USA; 4 Emergency Medicine, Arrowhead Regional Medical Center, Colton, USA

**Keywords:** pneumonia, monoclonal antibodies, casirivimab, imdevimab, coronavirus, covid-19

## Abstract

Infection by severe acute respiratory syndrome Coronavirus 2 (SARS-CoV-2) is known to have the highest mortality rate among the elderly and those with pre-existing medical conditions. Viral load has been directly correlated with increased risk of mortality in hospitalized patients. Once infected, symptoms first arise approximately six to seven days later followed by immunoglobulin M (IgM) antibodies appearing 8-12 days after onset of clinical symptoms. Recent studies have noted that the monoclonal antibody combination of casirivimab and imdevimab (REGN-COV2) effectively reduces viral load in infected seronegative non-hospitalized patients. However, research supporting the use of REGN-COV2 in an inpatient setting is limited. We present the case of a 45-year-old male with confirmed SARS-CoV-2 infection with moderate dyspnea and progressive worsening of his symptoms over a week period. The patient showed drastic improvement of his symptoms after a single low-dose regimen of REGN-COV2 infusion while admitted to the hospital and was subsequently discharged without further medical complications.

## Introduction

The ongoing Coronavirus disease of 2019 (COVID-19) pandemic is caused by severe acute respiratory syndrome Coronavirus (SARS-CoV-2), ribonucleic acid (RNA) betacoronavirus [1]. While most individuals infected with the virus have self-limiting symptoms, the mortality rate is high among the elderly and those with pre-existing medical conditions, including hypertension, cardiovascular disease, diabetes, chronic lung disease, and cancer. [1] Pulmonary infection with SARS-CoV-2 may be categorized into four stages of infection, pneumonia, complications, and exitus or healing [1]. The development of COVID-19 pneumonia is a more severe and complicated disease process characterized by massive pulmonary viral invasion and subsequent endogenous hyper-immune response [1]. Viral entry into cells is mediated by the SARS-CoV-2 spike glycoprotein’s interaction with angiotensin-converting-enzyme-2 (ACE2) receptor, which is commonly expressed in the lower respiratory tract [1]. Infected individuals show symptoms at an average of six days post-infection [1]. Subsequently, immunoglobulin M (IgM) antibodies appear approximately 8-12 days after onset of infection, and immunoglobulin G (IgG) predominates at approximately week 12 [1,2]. Furthermore, high viral loads have been noted to correlate with higher rates of death among hospitalized patients [3].

While there is no specific treatment available for COVID-19, recent data have suggested that monoclonal antibodies (mAbs) may play a vital role in reducing the viral load [4-7]. A few recent studies noted that one such therapeutic cocktail of mAbs is the combination of casirivimab and imdevimab (REGN-COV2) has been shown to effectively reduce viral load in infected seronegative non-hospitalized patients [4-6]. This cocktail specifically targets two distinct regions of the SARS-CoV-2 spike glycoprotein [4-6]. The recent Emergency Use Authorization (EUA) guidelines approved the use of REGN-COV2 in mild-to-moderate COVID-19 patients with a high risk for hospitalization or progression to severe disease [4,7]. While the therapeutic use of REGN-COV2 has been studied in outpatient care, there have been little to no reported cases of administration of this cocktail in an inpatient hospital setting. Here, we present a case of a patient with progressively worsening COVID-19 symptoms, who rapidly improved after REGN-COV2 treatment while admitted to the hospital.

## Case presentation

A 45-year-old male who tested positive for SARS-CoV-2 was presented to the emergency department (ED) with persistent non-productive cough, severe dyspnea, fever, chills, and intermittent diarrhea. His comorbidities include diabetes mellitus, hypertension, and morbid obesity (BMI 44.7 kg/m^2^). The patient's symptoms started eight days prior to his presentation to the ED. He experienced gradual worsening dyspnea at rest and daily fevers up to 105 °F. He denied loss of taste or smell. He also denied any history of alcohol, tobacco, or illicit drug use. On presentation, his vital signs included a blood pressure of 127/70 mmHg, respiratory rate of 24 per minute, fever of 103.6 °F, and oxygen saturation of 89% on room air. His physical examination was notable for tachypnea and bibasilar crackles.

**Figure 1:**
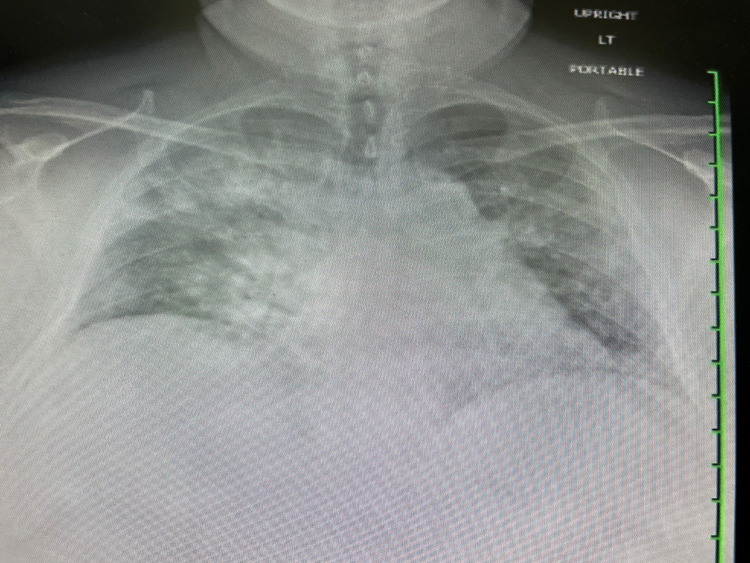
Diffuse bilateral airspace opacities noted on the chest X-ray

Chest X-ray revealed diffuse bilateral airspace opacities most prominent in the right upper lobe (Figure 1). Laboratory testing was significant for leukocytosis of 12,000 cells/µL, elevated absolute neutrophil count of 10,400 cells/µL (normal range =2500-7500 cells/µL), elevated D-dimer of 415 ng/mL (normal range <250 ng/mL), and elevated ferritin of 3329 µg/L (normal range = 20-300 µg/L). Serology was negative for antibodies to SARS-CoV-2 via an enzyme chemiluminescence assay (manufactured by Roche-Elecsys, Indianapolis, IN).

During the initial resuscitation in the ED, the patient’s oxygen saturation improved to 94% on 4 L/min of supplemental oxygen via nasal cannula. Additionally, he was given a single dose of dexamethasone 10 mg intravenous (IV), remdesivir 200 mg IV, and enoxaparin 40 mg subcutaneously (SQ) in the ED. After admission to the inpatient unit, he was continued on once-daily administration of dexamethasone 6 mg IV, remdesivir 100 mg, and enoxaparin 40 mg SQ.

Due to the patient’s complicated medical history, comorbidities, and risk of progressing to severe COVID-19 pneumonia, pulmonology/critical care and infectious disease specialists were consulted and recommended the administration of REGN-COV2. The consideration for IV infusion of REGN-COV2 via off-label use was based on the recently reported studies suggesting a significant reduction in viral load for seronegative patients on low-flow supplemental oxygen [4,7]. The risks, benefits, and alternatives for the antibody cocktail infusion were discussed with the patient, and he ultimately consented to the recommended treatment. Subsequently, he received a 2.4 g IV infusion of the antibody cocktail the evening of the first day of hospitalization. On the second day, his respiratory rate normalized, and his supplemental oxygen requirements decreased to 2 L/min. By the third day of his hospital course, the patient was only intermittently requiring supplemental oxygen while ambulating. The patient was ultimately discharged home with supplemental oxygen to be used as needed after five days of hospitalization. The patient experienced no further complications since his discharge from the hospital.

## Discussion

The combination of casirivimab and imdevimab (REGN-COV2) for the treatment of COVID-19 is based on the EUA of the United States Food and Drug Administration (FDA) for the care of patients in the outpatient setting [4-7]. Monoclonal antibodies bind to specific surface proteins on SARS-CoV-2 to neutralize its pathogenic effects [4,5]. Although alternative methods, such as convalescent plasma, can provide patients with antibodies that may aid in recovery, Deb et al. noted that monoclonal antibodies are safer and more specific in targeting SARS-CoV-2 surface proteins [6]. REGN-COV2 works optimally in lowering viral loads in patients who are serum antibody-negative at baseline [4]. This is supported by the findings of Weinrich et al. who demonstrated that patients who were not seronegative at baseline had a 2-log greater viral load at day seven post-infusion [4]. Two recent studies reported that a combination of the two monoclonal antibodies is more effective than a single monoclonal antibody in the treatment of the COVID-19 disease [8,9]. The efficacy of REGN-COV2 is likely due to casirivimab and imdevimab both targeting the spike protein on distinctly separate epitopes [4,5,10]. Baum et al. further suggested that the combination of the cocktail in REGN-COV2 prevents rapid mutational escape, which is an important consideration as mutant SARS-CoV-2 strains arise globally [9].

The expansion to inpatient use of REGN-COV2 may be beneficial to many high-risk individuals. In two studies that examined patients who were hospitalized for COVID-19, 17.8% and 28.4% of patients were intubated due to disease progression [11,12]. These studies demonstrate the high prevalence of invasive mechanical ventilation seen in COVID-19 patients who are unable to reduce progression to severe COVID-19. In this case, the patient received the monoclonal antibody cocktail during his hospitalization and made a rapid recovery, which was evidenced by the resolution of his tachypnea, fevers, and requirements for continuous supplemental oxygen. Current evidence in the literature suggests that two groups in the outpatient setting may benefit from receiving the REGN-COV2 antibody cocktail [3-5]. The two groups include patients who are unable to elicit an immune response and those who have not received a COVID-19 vaccination [3-5]. At the time of our patient’s presentation, the COVID-19 vaccine had just become available to the United States. He had not received a COVID-19 vaccine and was seronegative on initial laboratory studies. Because he was moderately symptomatic, positive for SARS-CoV-2 by reverse transcriptase-polymerase chain reaction (manufactured by PerkinElmer, Waltham, MA), and had multiple medical comorbidities, he was at high risk for progressing to severe COVID-19. After consulting infectious disease and pulmonology/critical care specialists and assessing the patient’s clinical prognosis, the decision was made to administer the REGN-COV2 cocktail as an off-label therapy with the prior consent of the patient. To minimize the risk for infusion reactions, the patient was given the low-dose formulation at 2.4 g single IV infusion over a period of 60 minutes, rather than the alternative single IV infusion at 8 g over 60 minutes. This method of infusion is recommended by the FDA and was also supported by Regeneron Pharmaceuticals’ initial trial, which reported improved clinical outcomes in the appropriate high-risk candidates [7]. In this case, the patient did not show any adverse reaction to the low-dose infusion therapy protocol. Given our patient’s successful recovery from his severe symptoms, there may be additional opportunities in utilizing the REGN-COV2 antibody cocktail for hospitalized patients as well. However, at this point, the administration of the cocktail should be reserved for those who fulfill the current criteria outlined by the FDA of mild-to-moderate COVID-19 disease, age ≥ 12, weight ≥ 40 kg, and high risk for hospitalization or progression to severe COVID-19 [4,5,7].

Recent studies suggest the use of monoclonal antibodies in the management of COVID-19 in seronegative patients [4-6,8,10]. Furthermore, two studies, in particular, support the use of REGN-COV2 in non-hospitalized patients who meet the clinical criteria for administration [4,5]. The clinical course of our patient supports the observations by Weinrich et al., who demonstrated that maximal viral load reduction occurred 48 hours after initiation of treatment [4]. This theory suggests that the low-dose form of REGN-COV2 is efficacious in reducing symptoms in moderate COVID-19 and likely reduced viral loads [4]. In addition, this report showcases the critical cooperation that was made between a multidisciplinary hospital care team and the patient and his family to improve the outcome and the course of his disease. The decision to administer the antibody cocktail was based on the evaluation of the critical stage of the patient’s clinical status accompanied by his risk factors. In addition, he met all of the criteria for REGN-COV2 administration except for being non-hospitalized. As a result, based on existing preliminary evidence, the patient, in this case, was an optimum candidate for the REGN-COV2 infusion. Future studies should evaluate the efficacy of monoclonal antibody cocktails in hospitalized patients requiring supplemental oxygen and further analyze the risks and benefits of low versus high doses of REGN-COV2.

## Conclusions

This case helps elucidate the potential benefits of inpatient administration of REGN-COV2 in the management of seronegative patients with a diagnosis of COVID-19. Clinicians may consider the use of monoclonal antibody cocktail infusions in hospitalized and outpatient care for patients at risk for progression to severe disease.
